# Two-year prevalence study of hospital-acquired bloodstream infections in university hospital Trnava, Slovakia

**DOI:** 10.1186/2047-2994-4-S1-P203

**Published:** 2015-06-16

**Authors:** M Garabasova, JS Brnova, M Betkova, A Streharova

**Affiliations:** 1 Department of Public Health, School of Health Sciences and Social Work, Trnava University, Trnava, Slovakia; 2 Department of Laboratory Medicine, School of Health Sciences and Social Work, Trnava University, Trnava, Slovakia; 3 Laboratory of Molecular Microbiology, St. Elisabeth University, Bratislava; 4 Department of Nursing, Constantine the Philosopher University, Nitra, Slovakia

## Introduction

In Slovakia, surveillance data of all health care associated infections, especially bloodstream infections (BSIs) are underestimated. Moreover, reporting methodology of BSIs in the national epidemiologic system is based on ICD code (septicemia).

## Objectives

The aim of this study was to assessed the prevalence of hospital-acquired bloodstream infections (HA – BSIs) and also to promoted HA – BSIs surveillance at hospital level in University hospital Trnava.

## Methods

This hospital-based prevalence study was conducted at the Trnava University hospital (618-bed; approx. 25 000 patients per year). We reviewed data from all consecutive positive blood cultures from microbiology laboratory database between 1 January 2013 and 31 December 2014. Hospital onset BSIs were defined as the first positive blood culture occurring more than 48 hours post-admission and were classified on ECDC criteria. Data were analysed using R-project, Fisher’s exact test or the chi-square test and differences between groups were considered to be significant when P-value < 0,05.

## Results

For the purposes of this study we analysed only laboratory-confirmed, hospital-acquired BSIs (n=256; 61,0% of all reported BSIs). The mean age of patients was 63,5 ±14 years and 59,4% (152) were men. The incidence of HA - BSI was 6,5/1000 admission (3,8 per 1000 admission in non-ICU patients vs. 34,0 per 1000 admission in ICU patients). A total 46,9% (120) occurred in intensive care units and 56, 7% (34) were catheter-related infections with an estimated rate 4 CRBSIs/1000 central - line days. In - hospital case fatality rate was 36,7% (43,3% in ICU vs. 23,5% in non - ICU; P=0.001). The most common pathogens were *Enterobacteriaceae* (42%), followed by coagulase-negative staphylococci and *Staphylococcus aureus* (15,2% for both) and *Pseudomonas aeruginosa* (11,6%).

## Conclusion

In this study, BSIs related to catheter, which are a leading preventable infectious complication of health care, occurred with high prevalence in our hospital. In near future, we required to establish better surveillance of HA-BSIs at the national level and implementation bundle care strategy for prevention of catheter-related BSIs at the local level.

## Disclosure of interest

None declared.

